# The Effect of Strict Volume Control Assessed by Repeated Bioimpedance Spectroscopy on Cardiac Function in Peritoneal Dialysis Patients

**DOI:** 10.1038/s41598-019-53792-0

**Published:** 2019-11-27

**Authors:** Yu Ah Hong, Hye Eun Yoon, Bum Soon Choi, Seok Joon Shin, Yong-Soo Kim, So Young Lee, Sang-Ho Lee, Su Hyun Kim, Eun Young Lee, Sug Kyun Shin, Young Joo Kwon, Jeong Ho Kim, Yoon Kyung Chang, Suk Young Kim, Ji Eun Kim, Shin Young Ahn, Gang Jee Ko

**Affiliations:** 1 Department of Internal Medicine, College of Medicine, The Catholic University of Korea, Seoul, Republic of Korea; 2 Department of Internal Medicine, Eulji University School of Medicine, Seoul, Republic of Korea; 3 Department of Internal Medicine, Kyung Hee University Medical School, Seoul, Republic of Korea; 4 Department of Internal Medicine, Chung-Ang University College of Medicine, Seoul, Republic of Korea; 5 Department of Internal Medicine, Soonchunhyang University Cheonan Hospital, Cheonan, Republic of Korea; 6 Department of Internal Medicine, National Health Insurance Service Ilsan Hospital, Goyang, Republic of Korea; 7 Department of Internal Medicine, Korea University School of Medicine, Seoul, Republic of Korea

**Keywords:** Outcomes research, Peritoneal dialysis

## Abstract

Adequate fluid management plays an important role in decreasing cardiovascular risk in peritoneal dialysis (PD) patients. We evaluated whether strict volume control monitored by bioimpedance spectroscopy (BIS) affects cardiac function in PD patients. This study is a secondary analysis of a multicentre, prospective, randomized, controlled trial. Fluid overload was assessed by the average overhydration/extracellular water (OH/ECW) at baseline, 6 months and 12 months. Patients were categorized as time-averaged overhydrated (TA-OH/ECW ≥15%) or normohydrated (TA-OH/ECW <15%), and echocardiographic parameters were compared between groups. Among a total of 151 patients, 120 patients exhibited time-averaged normohydration. Time-averaged overhydrated patients had a significantly higher left atrial (LA) diameter and E/e′ ratio and a lower left ventricular (LV) ejection fraction at 12 months than time-averaged normohydrated patients. LA diameter, end-systolic volume and end-diastolic volume were decreased at 12 months compared to baseline in time-averaged normohydrated patients only. TA-OH/ECW was independently associated with ejection fraction at 12 months (β = −0.190; p = 0.010). TA-OH/ECW, but not OH/ECW at 12 months, was an independent risk factor for LV dysfunction (odds ratio 4.020 [95% confidence interval 1.285–12.573]). Overhydration status based on repeated BIS measurements is an independent predictor of LV systolic function in PD patients.

## Introduction

Cardiovascular disease is the leading cause of mortality and morbidity in patients with chronic kidney disease (CKD) and end-stage renal disease (ESRD)^1^. Fluid overload is directly linked to hypertension, increased arterial stiffness and left ventricular (LV) hypertrophy^2,3^ and is an independent predictor of cardiovascular risk and mortality in ESRD patients^4–7^. Therefore, adequate fluid management is a key strategy to decrease cardiovascular risk in peritoneal dialysis (PD) patients. Chronic fluid overload occurs frequently in PD and non-dialysis CKD or haemodialysis (HD). Indeed, the European Body Composition Monitoring (EuroBCM) study demonstrated that more than 50% of PD patients were overhydrated, and severe fluid overload was present in 25.2% of PD patients^8^.

Bioimpedance spectroscopy (BIS) is a simple and noninvasive technique for measuring body composition and has been proposed as an effective objective method to assess and monitor the volume status^9^. Few randomized controlled trials have demonstrated that BIS-guided fluid management leads to LV mass index regression, decreased blood pressure (BP), improved arterial stiffness, and overall increased survival in maintenance HD patients^10,11^. However, only one randomized controlled trial demonstrated that fluid management guided by BIS significantly decreased BP in PD patients^12^. Recent randomized controlled studies have revealed that BIS-guided fluid management did not benefit volume control, residual renal function preservation, or cardiovascular parameters in PD patients^13,14^. Therefore, the usefulness of BIS measurement for the assessment of fluid balance has been inconclusive in PD patients.

The effects of strict volume control on cardiac outcomes and survival in dialysis patients have been reported in many previous studies^15–17^. However, traditional approaches, such as cardiothoracic ratio, BP, or brain natriuretic peptide, were not sensitive enough to detect subtle changes in fluid status. Most studies that evaluated the association between fluid overload measured by BIS and echocardiographic parameters in CKD patients were cross-sectional analyses^3,18,19^. Therefore, the effect of overhydration status assessed by BIS measurements on cardiac function over time remains unclear. The aim of this study was to elucidate whether overhydration status assessed by repeated BIS measurements for one year influences cardiac function in non-anuric PD patients.

## Results

### Study population

**Figure 1:**
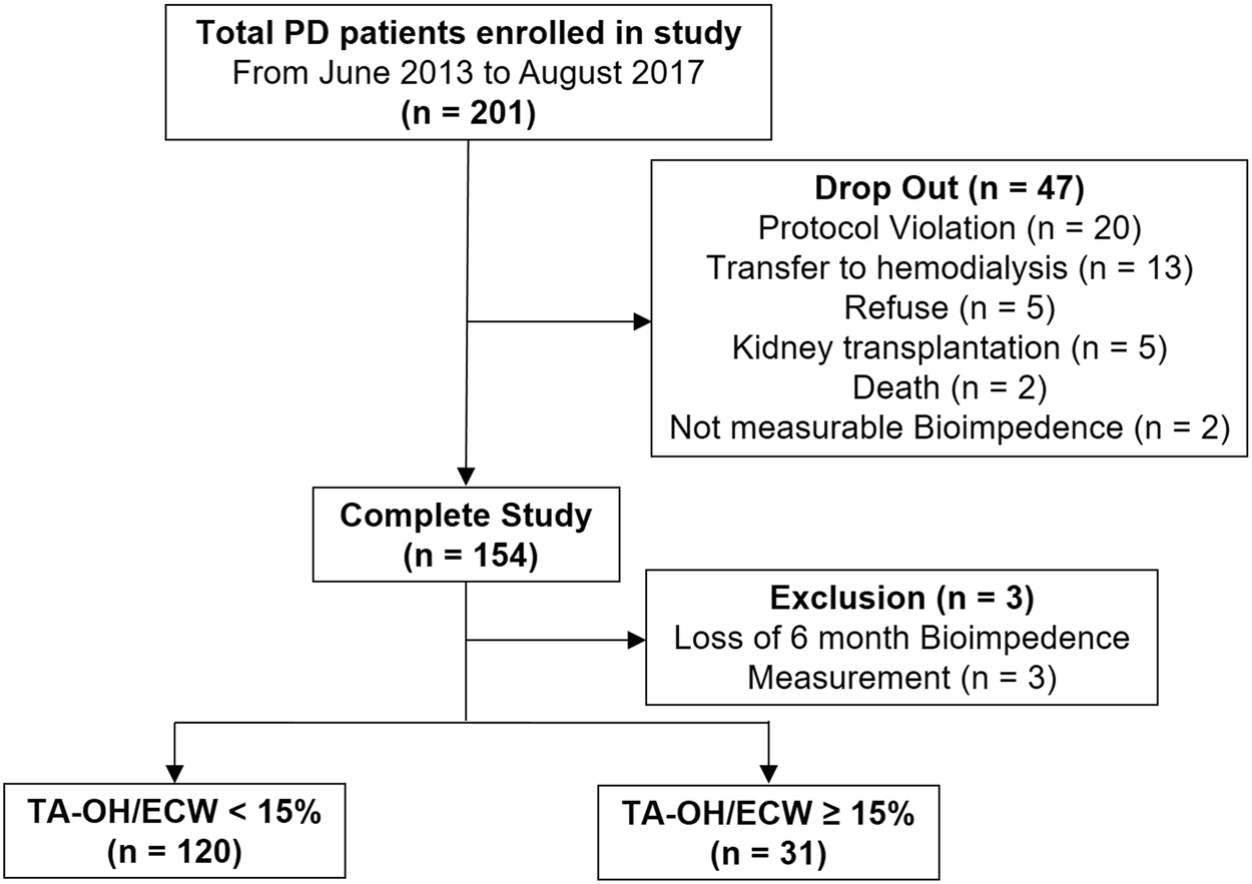
Study design and flow chart of study participants.

**Figure 2:**
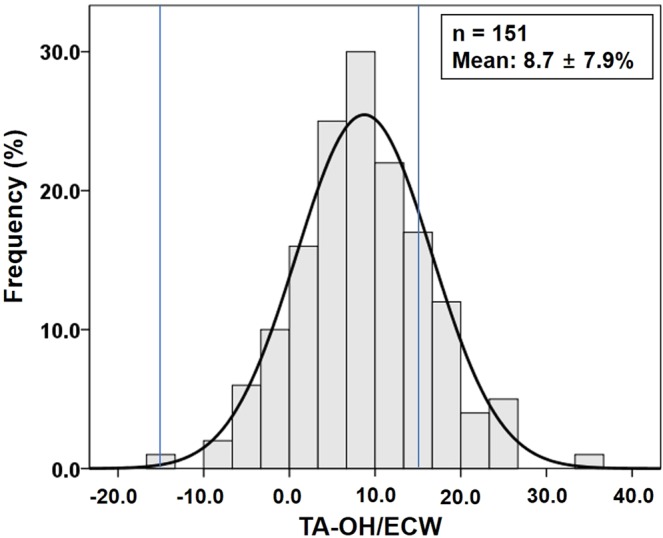
Distribution of average relative overhydration over one year (TA-OH/ECW) in 151 PD patients, ranging between −14% and 35% (8.7 ± 7.9%; median 8.6%).

Figure 1 depicts the study design and contains a flowchart of the study participants. In the original randomized controlled trial, a total of 201 participants were enrolled and randomly assigned to the BIS-guided or the control group. In total, 154 PD patients completed the study (Fig. 1). We excluded three patients who had no BIS measurement results for 6 months. Therefore, 151 patients (53.9 ± 12.1 years, 54.3% male) were ultimately included in this study. The mean value of time-averaged overhydration/extracellular water (TA-OH/ECW), the average of overhydration/extracellular water (OH/ECW) at baseline, 6 months and 12 months, was 8.7 ± 7.9% (Fig. 2). Thirty-one patients (20.5%) were categorized into the time-averaged overhydrated group (TA-OH/ECW ≥15%), and 120 (79.5%) patients were categorized into the time-averaged normohydrated group (TA-OH/ECW <15%).

### Patient characteristics at baseline and 12 months

Table 1 shows the study population characteristics at baseline and 12 months. The patients in the two groups were similar regarding age, sex, body mass index (BMI), and presence of hypertension. However, there were more patients with diabetes mellitus (DM) and diabetic kidney disease in the time-averaged overhydrated group. Patients with time-averaged overhydration were taking more anti-hypertensive agents and had a higher ratio of dialysate to serum creatinine (D/P creatinine) from the 4-hr peritoneal equilibrium test (PET) compared to individuals with time-averaged normohydration at both baseline and 12 months. PD duration and daily urine output were similar between the groups. Pulse pressure, but not systolic and diastolic BP, was higher in time-averaged overhydrated patients at baseline and 12 months. Patients with time-averaged overhydration had significantly lower serum albumin levels at baseline and 12 months. Haemoglobin was not different between the two groups at baseline but was significantly decreased in time-averaged overhydrated patients at 12 months. Additionally, serum glucose was markedly higher in time-averaged overhydrated patients than normohydrated patients at baseline but was not significantly different between the two groups at 12 months.

**Table 1:** The comparison of clinical and laboratory parameters between study groups at baseline and the end of study.

	Baseline			12 months		
	TA-OH/ECW <15% (n = 120)	TA-OH/ECW ≥15% (n = 31)	p value	TA-OH/ECW <15% (n = 120)	TA-OH/ECW 15% (n = 31)	p value
Age	53.5 ± 12.8	55.8 ± 9.0	0.342			
Sex (male) (%)	66 (55.0)	16 (51.6)	0.736			
BMI (Kg/m^2^)	24.5 ± 3.4	23.9 ± 4.0	0.419			
DM (%)	43 (35.8)	26 (83.9)	<0.001			
Hypertension (%)	96 (80.0)	26 (83.9)	0.626			
Cause of ESRD (%)			0.001			
DM (%)	38 (31.7)	22 (71.0)				
Hypertension (%)	39 (32.5)	3 (9.7)				
CGN (%)	12 (10.0)	2 (6.5)				
Others/Unknown (%)	31 (25.8)	4 (12.9)				
PD duration (months)	11.8 ± 21.0	11.3 ± 19.0	0.909			
D/P Cr at 4-hr PET	0.61 ± 0.12	0.67 ± 0.16	0.037	0.60 ± 0.11	0.66 ± 0.13	0.024
No. of anti-hypertensive agents	2.0 ± 1.5	2.7 ± 1.6	0.025	1.3 ± 1.5	2.0 ± 1.4	0.008
Urine output (L/day)	1.00 ± 0.47	1.24 ± 1.71	0.444	0.83 ± 0.48	0.81 ± 0.63	0.855
Systolic BP (mmHg)	132.1 ± 25.6	139.7 ± 24.8	0.143	133.6 ± 19.7	142.0 ± 25.8	0.100
Diastolic BP (mmHg)	79.4 ± 13.2	76.2 ± 11.0	0.220	79.7 ± 10.7	77. 8 ± 11.0	0.377
Pulse pressure (mmHg)	52.7 ± 19.0	63.5 ± 19.3	0.006	53.9 ± 15.4	64.2 ± 18.9	0.002
Laboratory findings						
Hemoglobin (g/dL)	11.1 ± 1.2	10.8 ± 1.5	0.241	11.1 ± 1.6	10.3 ± 1.3	0.029
Glucose (mg/dL)	122.1 ± 56.7	159.4 ± 89.7	0.034	128.1 ± 70.1	153.2 ± 77.6	0.084
Serum Creatinine (mg/dL)	8.1 ± 2.7	7.5 ± 3.1	0.289	9.5 ± 3.4	9.1 ± 3.3	0.471
Serum Albumin (g/dL)	3.7 ± 0.4	3.3 ± 0.5	<0.001	3.7 ± 0.4	3.4 ± 0.5	<0.001
Echocardiographic parameters						
LA diameter (mm)	38.2 ± 6.2	40.8 ± 7.2	0.052	36.7 ± 6.1	39.4 ± 6.7	0.036
LA volume (ml)	48.3 ± 20.8	51.5 ± 19.4	0.628	45.8 ± 22.9	49.4 ± 22.4	0.477
LVESd (mm)	32.9 ± 9.7	34.1 ± 6.9	0.512	31.6 ± 10.6	34.5 ± 9.4	0.165
LVEDd (mm)	50.2 ± 11.1	51.1 ± 9.5	0.676	48.7 ± 11.4	50.7 ± 8.5	0.359
LV mass index (g/m^2^)	128.6 ± 69.9	140.4 ± 61.9	0.395	121.1 ± 56.1	120.1 ± 46.9	0.926
ESV (ml)	38.5 ± 21.7	39.0 ± 18.6	0.904	32.2 ± 15.1	37.5 ± 18.6	0.107
EDV (ml)	101.8 ± 41.0	100.7 ± 35.8	0.885	83.9 ± 31.7	93.7 ± 34.7	0.145
EF (%)	62.8 ± 10.1	60.0 ± 7.8	0.155	61.6 ± 6.0	58.8 ± 7.9	0.034
e′ velocity (cm/sec)	5.7 ± 2.8	3.7 ± 2.9	0.001	5.9 ± 4.7	3.7 ± 2.6	0.010
E/A	0.88 ± 0.44	0.77 ± 0.22	0.047	0.88 ± 1.2	0.67 ± 0.14	0.347
E/e′	11.7 ± 11.1	14.9 ± 6.0	0.131	10.3 ± 4.4	13.3 ± 5.0	0.004

^*^Abbreviation: BMI, body mass index; BP, blood pressure; CGN, chronic glomerulonephritis; DM, diabetes mellitus; D/P Cr, dialysate/peritoneal fluid creatinine ratio; e′, early diastolic mitral annular tissue velocity; E/A, early diastolic mitral inflow velocity/late diastolic mitral inflow velocity; EDV, end-diastolic volume; E/e′, early diastolic mitral inflow velocity/early diastolic mitral annular tissue velocity, EF, ejection fraction; ESRD, end-stage renal disease; ESV, end-systolic volume; LA, left atrial; LV, left ventricular; LVEDd, left ventricular end-diastolic diameter; LVESd, left ventricular end-systolic diameter; PD, peritoneal dialysis; PET, peritoneal equilibrium test.

Comparisons of echocardiographic parameters between the time-averaged overhydrated group and the normohydrated group at baseline and 12 months are also found in Table 1. At baseline, the time-averaged overhydrated group exhibited a lower e′ velocity and E/A ratio than did the normohydrated group (p = 0.001 and p = 0.047, respectively). LA volume, LV end-systolic diameter (LVESd), LV end-diastolic diameter (LVEDd), LV mass index, end-systolic volume (ESV) and end-diastolic volume (EDV) were not different between the two groups at either baseline or 12 months. However, the LA diameter and E/e′ ratio were significantly higher (p = 0.036 and p = 0.004, respectively), and the LV ejection fraction (EF) and e′ velocity were lower in overhydrated patients compared to normohydrated patients (p = 0.034 and p = 0.010, respectively) at 12 months.

### Differences in BIS parameters between overhydrated and normohydrated patients at baseline and 12 months

**Figure 3:**
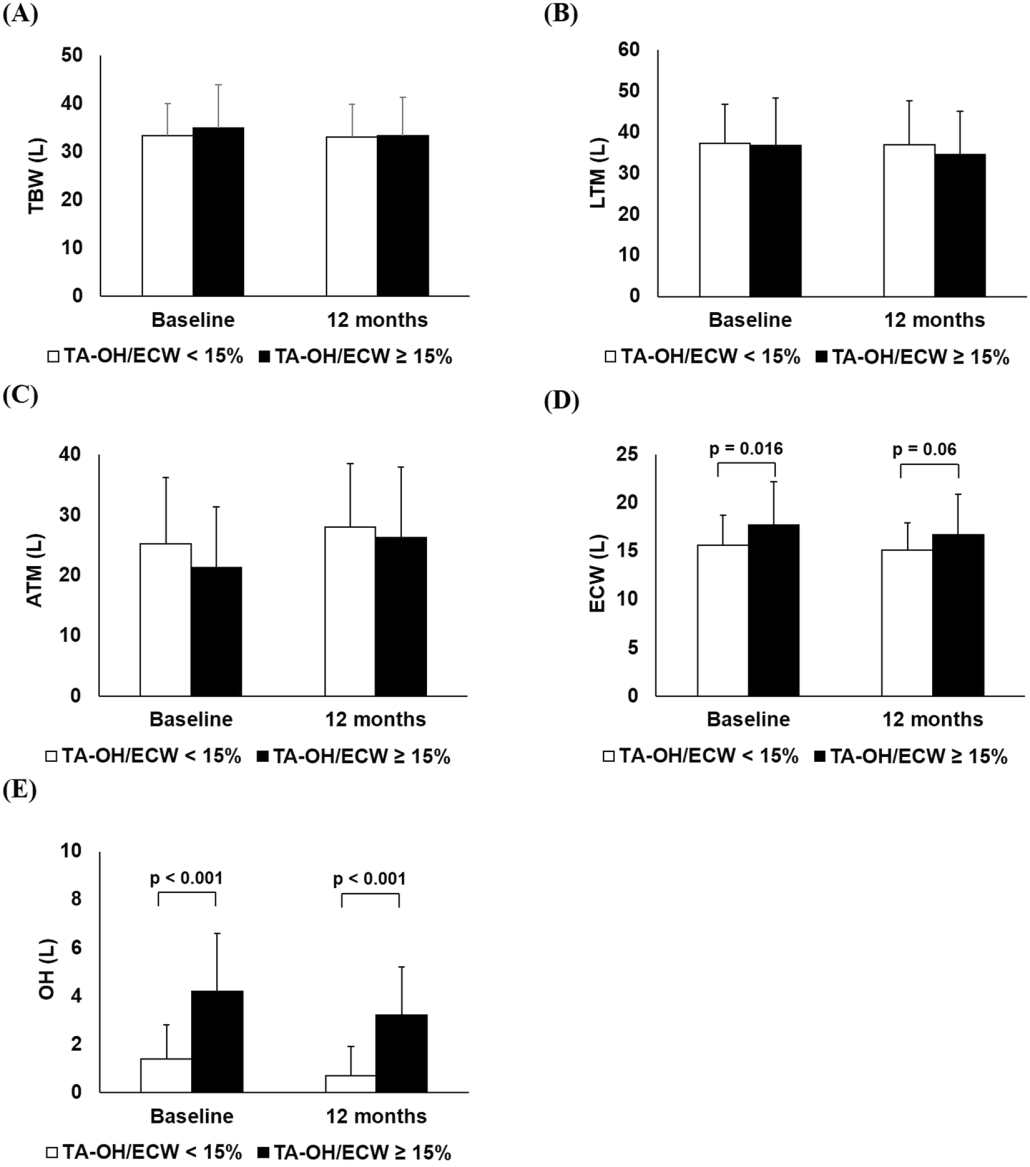
The comparison of changes of BIS Parameters between patients with TA-OH/ECW ≥15% and TA-OH/ECW <15% at baseline and the end of study. (**A**) Total body water, (**B**) Lean tissue mass, (**C**) Adipose tissue mass, (**D**) Extracellular water (ECW), and (**E**) Overhydration (OH).

Among body composition parameters measured, total body water (TBW), lean tissue mass (LTM) and adipose tissue mass (ATM) were not significantly different between the time-averaged overhydrated group and normohydrated group at either baseline or 12 months (Fig. 3A–C). Baseline extracellular water (ECW) and overhydration (OH) were further increased in the time-averaged overhydrated group compared to the normohydrated group (Fig. 3D,E, p = 0.016 and p < 0.001, respectively). ECW was no longer significantly different between the two groups at 12 months (Fig. 3D, p = 0.06), but OH remained higher in the time-averaged overhydrated group at 12 months (Fig. 3E, p < 0.001, respectively).

### Echocardiographic parameters associated with time-averaged relative hydration status

As shown in Table 2, linear regression analyses were performed to identify associations between echocardiographic parameters at 12 months and TA-OH/ECW. LA diameter, LA volume, LVESd, ESV, and EDV at the end of study were all positively associated with TA-OH/ECW (LA diameter: β = 0.338; p < 0.001, LA volume: β = 0.197; p = 0.032, LVESd: β = 0.220; p = 0.007, ESV: β = 0.240; p = 0.004, and EDV: β = 0.207; p = 0.012), and EF and e′ velocity were negatively associated with TA-OH/ECW in the univariable linear regression analysis (EF: β = −0.230; p = 0.004 and e′ velocity: β = −0.215; p = 0.008). Using the multivariable linear regression, TA-OH/ECW was independently negatively associated with only EF (β = −0.190; p = 0.010) after adjusting for age, PD duration, BMI, baseline OH, pulse pressure, haemoglobin, albumin, glucose, D/P creatinine and echocardiographic parameters that displayed statistical significance in the univariable analysis.

**Table 2:** Univariable and multivariable linear regressions for time-averaged relative hydration status and echocardiographic parameters at the end of study.

	Univariable			Multivariable		
	β	t	p value	β	t	p value
12-month LA diameter (mm)	0.338	4.388	<0.001	0.059	0.824	0.412
12-month LA volume (ml)	0.197	2.165	0.032	−0.059	−0.729	0.468
12-month LVESd (mm)	0.220	2.743	0.007	0.087	1.522	0.132
12-month LVEDd (mm)	0.132	1.614	0.109	—	—	—
12-month LV mass index (g/m^2^)	0.081	0.983	0.327	—	—	—
12-month ESV (mL)	0.240	2.958	0.004	−0.252	−1.352	0.180
12-month EDV (mL)	0.207	2.531	0.012	0.116	0.683	0.497
12-month EF (%)	−0.230	−2.890	0.004	−0.190	−2.618	0.010
12-month e′ velocity (cm/sec)	−0.215	−2.678	0.008	−0.003	−0.048	0.962
12-month E/A	−0.042	−0.515	0.607	—	—	—
12-month E/e′	0.151	1.855	0.066	−0.039	−0.675	−0.502

*Abbreviation: EDV, end-diastolic volume; E/e′, early diastolic mitral inflow velocity/early diastolic mitral annular tissue velocity; EF, ejection fraction; ESV, end-systolic volume; LA, left atrial; LV, left ventricular; LVEDd, left ventricular end-diastolic diameter; LVESd, left ventricular end-systolic diameter; TA-OH/ECW, time-averaged overhydration/extracellular water. The variables included in the multivariable linear regression model were age, PD duration, BMI, baseline OH, time-averaged pulse pressure, time-averaged hemoglobin, time-averaged albumin, time-averaged glucose, time-averaged D/P Creatinine at 4-hr PET and statistically significant variables among echocardiographic parameters in the univariable linear regression (p < 0.1). Dashes indicate that the variable did not enter the multivariable linear regression model.

### Changes in echocardiographic parameters between baseline and 12 months in time-averaged overhydrated and normohydrated patients

Table 3 shows the differences between baseline and 12-month echocardiographic parameters in the time-averaged overhydrated group and the normohydrated group. The LA volume and LV mass index were significantly decreased at 12 months compared to baseline in both the time-averaged overhydrated and normohydrated patients. However, LA diameter, ESV and EDV were significantly decreased in patients with time-averaged normohydration only (p = 0.014, p < 0.001, and p < 0.001, respectively), and the E/A ratio was significantly decreased at 12 months compared to baseline in patients with time-averaged overhydration only (p* = *0.034). EF was decreased at 12 months compared to baseline in patients with time-averaged overhydration; however, this difference was not statistically significant.

**Table 3:** The differences of echocardiographic parameters between baseline and the end of study in time-averaged overhydrated and normohydrated patients.

	TA-OH/ECW <15% (n = 120)			TA-OH/ECW ≥15% (n = 31)		
	Baseline	12 months	p value	Baseline	12 months	p value
LA diameter (mm)	38.2 ± 6.2	36.7 ± 6.1	0.014	40.8 ± 7.2	39.4 ± 6.7	0.305
LA volume (ml)	48.3 ± 20.8	45.8 ± 22.9	<0.001	51.5 ± 19.4	49.4 ± 22.4	0.001
LVESd (mm)	32.9 ± 9.7	31.6 ± 10.6	0.176	34.1 ± 6.9	34.5 ± 9.4	0.811
LVEDd (mm)	50.2 ± 11.1	48.7 ± 11.4	0.178	51.1 ± 9.5	50.7 ± 8.5	0.837
LV mass index (g/m^2^)	128.6 ± 69.9	121.1 ± 56.1	0.046	140.4 ± 61.9	120.1 ± 46.9	0.008
ESV (ml)	38.5 ± 21.7	32.2 ± 15.1	<0.001	39.0 ± 18.6	37.5 ± 18.6	0.488
EDV (ml)	101.8 ± 41.0	83.9 ± 31.7	<0.001	100.7 ± 35.8	93.7 ± 34.7	0.148
EF (%)	62.8 ± 10.1	61.6 ± 6.0	0.147	60.0 ± 7.8	58.8 ± 7.9	0.531
e′ velocity (cm/sec)	5.7 ± 2.8	5.9 ± 4.7	0.465	3.7 ± 2.9	3.7 ± 2.6	0.615
E/A	0.88 ± 0.44	0.88 ± 1.2	0.857	0.77 ± 0.22	0.67 ± 0.14	0.034
E/e′	11.7 ± 11.1	10.3 ± 4.4	0.188	14.9 ± 6.0	13.3 ± 5.0	0.144

*Abbreviation: e′, early diastolic mitral annular tissue velocity; E/A, early diastolic mitral inflow velocity/late diastolic mitral inflow velocity; EDV, end-diastolic volume; E/e′, early diastolic mitral inflow velocity/early diastolic mitral annular tissue velocity; EF, ejection fraction; ESV, end-systolic volume; LA, left atrial; LV, left ventricular; LVEDd, left ventricular end-diastolic diameter; LVESd, left ventricular end-systolic diameter.

### The effect of time-averaged overhydration status on LV systolic function at 12 months

To evaluate whether strict volume control during the one-year period was associated with LV systolic function at the end of the study, we performed the univariable and multivariable logistic regression analyses for EF at 12 months (Table 4). In the crude model, patients with time-averaged overhydration had an increased risk of LV systolic dysfunction, defined as EF < 55%, compared to patients with time-averaged normohydration as the reference category (odds ratio (OR) 2.863, 95% confidence interval (CI), [1.065–7.699], p = 0.037). In the multivariable analysis, time-averaged overhydration status was a powerful independent predictor of LV systolic dysfunction (OR 4.020, 95% CI [1.285–12.573], p* = *0.017) after adjusting for age, sex, presence of DM, cause of ESRD, PD duration, baseline OH, time-averaged D/P creatinine from 4-hr PET, time-averaged pulse pressure, time-averaged haemoglobin, time-averaged glucose, time-averaged albumin, LA diameter, LV mass index and E/e′ ratio at 12 months. However, overhydration status at 12 months did not significantly affect LV systolic function in the univariable and multivariable analyses.

**Table 4:** Univariable and multivariable logistic regression for the predictors of left ventricular systolic dysfunction at the end of study.

	Crude OR		Model 1		Model 2	
	OR (95% CI)	p value	OR (95% CI)	p value	OR (95% CI)	p value
12-month OH/ECW						
<15%	1 (Reference)		1 (Reference)		1 (Reference)	
≥15%	1.646 (0.582–4.654)	0.347	0.736 (0.173–3.119)	0.667	1.006 (0.213–4.738)	0.994
TA-OH/ECW						
<15%	1 (Reference)		1 (Reference)		1 (Reference)	
≥15%	2.863 (1.065–7.699)	0.037	4.556 (1.520–13.657)	0.007	4.020 (1.285–12.573)	0.017

*Abbreviation: CI, confidence interval; OH/ECW, overhydration/extracellular water; OR, odds ratio; TA-OH/ECW, time-averaged overhydration/extracellular water.

*Multivariable logistic regression for 12-month OH/ECW was adjusted for Model 1: age, sex, BMI, presence of DM, cause of ESRD, PD duration, baseline OH, 12-month pulse pressure, 12-month D/P creatinine at 4-hr PET, 12-month hemoglobin, 12-month glucose, 12-month albumin, and Model 2: model 1 + 12-month LA diameter, 12-month LV mass index, and 12-month E/e′ ratio.

*Multivariable logistic regression for time-averaged OH/ECW was adjusted for Model 1: age, sex, BMI, presence of DM, cause of ESRD, PD duration, baseline OH, time-averaged pulse pressure, time-averaged D/P creatinine at 4-hr PET, time-averaged hemoglobin, time-averaged glucose, time-averaged albumin, and Model 2: model 1 + 12-month LA diameter, 12-month LV mass index, and 12-month E/e′ ratio.

## Discussion

In this study, we demonstrated that time-averaged overhydrated PD patients, as assessed by repeated BIS measurements, had a higher LA diameter and E/e′ ratio and a lower EF than did time-averaged normohydrated PD patients at 12 months, and the time-averaged normohydrated status improved the volume-associated echocardiographic parameters after 12 months. Furthermore, time-averaged overhydration was significantly associated with decreased EF in regression analysis, even after adjusting for confounding variables. These findings suggest that hydration status assessed by repeated BIS measurements may be useful to maintain LV systolic function in PD patients.

Various previous studies have reported that fluid overload assessed by BIS is an independent risk factor for all-cause and cardiovascular mortality in dialysis patients^5,6,20–22^. Our original randomized controlled study investigated the clinical benefit of BIS-guided fluid management, targeting OH within ± 2 L of the normal range, over conventional management for one year. However, we did not demonstrate differences in clinical outcomes, such as residual renal function, 24-hr urine volume and echocardiographic parameters, between the control and BIS-guided groups^23^. The recently published COMPASS study also did not demonstrate any differences in clinical outcomes between the BIS-guided group and the control group^13^. There are several reasons for the unexpected results regarding the effect of BIS-guided fluid management in these studies. First, the enrolled PD patients all had well-preserved residual kidney function and relatively stable fluid status over the one-year period, even in the control group. Second, although the BIS information was not provided to the physicians, the enrolled patients in the control group had been assessed and managed fluid balance carefully, similar to those in the BIS-guided group, during the study period. Third, the study duration was relatively short and may not have been long enough to demonstrate the benefits of BIS-guided fluid management. These studies indicate that the careful assessment and treatment of fluid status *per se* is very important, regardless of BIS method use, in PD patients. Therefore, we focused on whether repeated monitoring of volume status using a BIS device had clinical benefits, particularly improved cardiac parameters, in PD patients.

We used the OH/ECW ratio instead of OH to define hydration status in consideration of the body size of the patients, and overhydration status was commonly defined as OH/ECW > 15%^24^. This definition of overhydration was attributed to a previous study by Wizemann *et al*.^6^. These authors demonstrated that a cutoff of 15% for OH/ECW was an independent predictor of survival in dialysis patients undergoing maintenance HD. Another recent study also showed that chronic fluid overload over a period of one year, which was defined as OH/ECW > 15% at baseline and 12 months, strongly predicted the risk of death and transfer to HD in prevalent PD patients^25^. However, these authors did not perform echocardiography and evaluate the cause of death or cardiovascular events, and the relationship between overhydration measured by BIS and cardiac function is difficult to determine. In the present study, we adopted TA-OH/ECW as the definition of hydration status to reflect the effect of longitudinal BIS measurement based on changes in hydration status and elucidated the association between overall fluid overload and cardiac function.

In this study, patients with time-averaged overhydration consisted of 20% of the total study population. These individuals were more likely to have DM, higher D/P creatinine via 4-hr PET, a higher number of anti-hypertensive medications, higher pulse pressure and glucose, and lower albumin than those of time-averaged normohydrated patients. Similar findings have also been reported in previous studies. Low serum albumin is an important determinant of overhydration in PD patients^26–28^. Overhydration may be due to reduced plasma filling, which can result from reduced oncotic pressure due to the significant peritoneal protein losses and decreases in serum albumin in PD patients^28^. Hydration status can also be affected by the rate of solute transfer across the peritoneal membrane. Rapid peritoneal solute transport, namely, high D/P creatinine, is associated with increased protein loss and excess dialysate reabsorption, leading to ultrafiltration problems and overhydration^29,30^. In addition, pulse pressure, known as arterial compliance, is independently associated with rapid peritoneal solute transport^31^, increased cardiovascular hospitalization, and increased mortality in PD patients^32^. Taken together, these complex clinical factors may contribute to fluid overload in PD patients.

The noteworthy finding of this study is to demonstrate the importance of continuous strict volume control on cardiac function and the usefulness of repeated BIS measurement for monitoring volume status in PD patients. We found that the time-averaged normohydrated group monitored by repeated BIS measurements had a significantly low LA diameter and E/e′ ratio at 12 months compared to those of the time-averaged overhydrated group and decreased volume-associated echocardiographic parameters, such as LA diameter, ESV and EDV, were observed after 12 months. Fluid overload can lead to increased cardiac workload, increased LV mass, and systolic and diastolic dysfunction caused by myocardial fibrosis^33^. To date, EF is the most frequently used parameter to define LV systolic function and an important prognostic marker of patients with cardiovascular diseases. In the present study, the mean EF value at baseline was not significantly different between the two groups, but EF in patients with time-averaged overhydration was significantly lower than that in patients with time-averaged normohydration at 12 months. In addition, TA-OH/ECW was significantly associated with EF and an independent predictor of LV systolic dysfunction at 12 months, whereas 12-month OH/ECW did not show an association with LV systolic dysfunction. Although the absolute differences of EF were small between the two groups and the mean EF value even in the overhydrated group was within normal ranges, the association between time-averaged fluid overload measured by BIS and the decrease in LV systolic function was observed in this study.

Several cross-sectional studies have reported that the LV mass index is positively correlated with fluid overload in HD patients^16,34,35^. However, we did not confirm the association between TA-OH/ECW and the LV mass index in this study. The LV mass index was not significantly different between the time-averaged overhydrated and normohydrated groups at baseline or 12 months. In contrast, the LV mass index was significantly decreased at 12 months compared to baseline in the time-averaged normohydrated group and in the overhydrated group. This discrepancy may be due to differences in study design, study length, and the definition of overhydration. Strict volume control through moderate salt restriction and diuretic use significantly improved the LV mass index after 3 years in PD patients and one year in HD patients^15,36^. These two previous studies were retrospective single-arm observation studies that did not compare the LV mass index between overhydrated and normohydrated patients. Therefore, more specific research for the relationship between fluid overload and the LV mass index in PD patients may be required.

Our study had some limitations. First, this observational study included both incident and prevalent PD patients, and 70% of the enrolled patients were within one year of initiating PD. We cannot exclude residual confounding factors given the observational study design. Second, the number of patients was relatively small, and the study period was relatively short. Third, despite the multicentre nature of this study, the participants were all Korean PD patients. Therefore, these results may not be applicable to other PD populations. Fourth, EF measured by conventional echocardiography has been shown to be rather insensitive to the detection of LV systolic dysfunction because of intra- and inter-observer variability^37^. Furthermore, LV hypertrophy and changes in LV structure in CKD patients could lead to LV systolic dysfunction despite normal EF^38,39^. In this respect, LV global longitudinal strain (GLS), the negative ratio of the maximal change in LV longitudinal length in systole to the original length as assessed by speckle tracking echocardiography, is effective in the early detection of LV systolic dysfunction in CKD patients with preserved EF^38,40^. A recent cross-sectional study showed that fluid overload measured by BIS was negatively correlated with GLS in pre-dialysis CKD patients^19^. Therefore, further studies using two-dimensional speckle tracking echocardiography may be needed to accurately elucidate the association between fluid overload and LV systolic dysfunction in dialysis patients. Despite these limitations, our study clearly demonstrates the clinical significance of regular BIS measurement for improving echocardiographic parameters in non-anuric PD patients.

In conclusion, strict volume control based on repeated measurements of BIS was an independent predictor of LV systolic function in non-anuric PD patients. Close monitoring by repeated BIS measurement and management to control volume could reduce overhydration-related morbidity and mortality. Further studies are needed to confirm whether adequate fluid management via repeated BIS measurements will lead to improved cardiovascular outcomes in PD patients with overhydration.

## Materials and Methods

### Study population and design

This study is a secondary analysis of a multicentre, prospective, randomized, controlled trial that was conducted for one year in Korea. Incident or prevalent PD patients from eight tertiary hospitals who were > 18 years old, had been treated for PD for at least three months and who had a daily urine output > 500 mL were enrolled. Patients who were not suitable for BIS measurements due to artificial devices or health conditions were excluded. This study was conducted according to the Declaration of Helsinki. The research protocol was approved by the Institutional Review Board of the Catholic University of Korea, Seoul St. Mary’s Hospital, the Institutional Review Board of the Catholic University of Korea, Incheon St. Mary’s Hospital, the Institutional Review Board of Eulji University School of Medicine, the Institutional Review Board of Kyung Hee University Medical School, the Institutional Review Board of Chung-Ang University College of Medicine, the Institutional Review Board of Soonchunhyang University Cheonan Hospital, and the Institutional Review Board of National Health Insurance Service Ilsan Hospital and the Institutional Review Board of Korea University School of Medicine, and informed consent was obtained from all participants.

The original study design included the comparison of clinical outcomes between patients with BIS-guided fluid management and those who managed the volume status by physical examinations and clinical assessment depending on symptoms and changes in body weight or BP. The patients were randomly allocated 1:1 to either the BIS or control group. In the control group, fluid was managed on the basis of physical examinations and clinical assessment. BIS measurements were also performed in the control group at baseline, 6 months and 12 months, but clinicians and participants were blinded to the results during the study period. The participants in the BIS group underwent monthly BIS-guided fluid management until OH reached within ± 2 L of normal range, and then BIS was performed every three months. All BIS results were provided to the clinicians and the participants. The prescriptions changed based on the BIS measurements to meet a target OH within ± 2 L of range.

The strategies of fluid management according to the results of BIS measurement or clinical assessment were as below: If patients were regarded in overhydrated status, the clinicians prescribed one or multiple following methods for fluid management as their judgment: (1) the restriction of dietary sodium intake, (2) the increase of diuretic dose or the use of other diuretic, and/or (3) the use of dialysate with higher glucose concentration. If patients were regarded in volume-depleted status, following either one or both were prescribed: (1) the reduction of diuretic dose or the discontinuation of diuretics and/or (2) the decrease of dialysate glucose concentration or the liberal intake of dietary sodium if the patient already underwent PD using the lowest dialysate glucose concentration. If patients were regarded in euvolemic status, the previous fluid management strategy was maintained. The prescriptions for fluid management were chosen based on the clinician’s judgment, and multiple prescriptions were permitted.

In this study, we defined the average of OH/ECW at baseline, 6 months and 12 months as the TA-OH/ECW. Patients were categorized into two groups based on relative hydration status during the one-year study period: time-averaged normohydrated and overhydrated groups. We compared changes in echocardiographic parameters from baseline to 12 months between the time-averaged overhydrated and normohydrated groups.

### Bioimpedance parameters

The Body Composition Monitor method (BCM, Fresenius Medical Care, Germany) was performed by trained practitioners at each centre. The BIS uses alternating electric currents at 50 frequencies between 5 and 1000 kHz. Measurement of BIS was performed by placing electrodes on one hand and one foot and entering the information of current height and body weight data into the device. Because BIS does not measure sequestered fluid in the trunk, indwelling intra-abdominal dialysate fluid does not influence the readings of hydration status^41^. Therefore, participants performed BIS under a dwelling state of dialysate. TBW, LTM, ATM, ECW and OH were identified by the measured BIS data. The OH is the difference between the expected ECW under normal physiological conditions and the actual ECW^42^. Because specific OH values vary in clinical relevance according to the body size of the patient, the OH/ECW ratio reflects relative the hydration status and can be a more accurate indicator of fluid overload than OH alone.

### Echocardiographic parameters

All patients underwent echocardiographic evaluation at baseline and the end of the study. A two-dimensional guided M-mode echocardiography was performed by cardiologists at each centre who did not know the clinical and laboratory information for the patient. LVESd or LVEDd were measured in the M-mode LV dimension with the short axis view at end-systole and end-diastole. LV mass was calculated using the Devereux formula and the LV mass index was determined from the LV mass/body surface area (g/m^2^). EF, ESV, EDV, LA diameter and LA volume were determined from apical two- and four-chamber views by Simpson’s biplane formula. Mitral inflow velocities and LV myocardial tissue velocities were determined using pulsed-wave Doppler and tissue Doppler imaging. We measured the peak early diastolic mitral inflow velocity (E), peak late diastolic mitral inflow velocity (A), and early diastolic mitral annular tissue velocity (e′). The ratio of peak early diastolic mitral inflow velocity to peak early diastolic mitral annular tissue velocity (E/e′) and the ratio of peak early diastolic mitral inflow velocity to peak late diastolic mitral inflow velocity (E/A) were calculated.

### Clinical and laboratory parameters

Physical examinations were performed at monthly visits. BP was determined as the average of two measurements every five minutes using a single calibrated BP device at each centre. BMI was defined as the formula of body weight/height^2^ (kg/m^2^). The presence of DM and hypertension, the cause of ESRD, and PD duration were collected from medical records. Peritoneal membrane characteristics were determined by the 4-hr PET, and a D/P creatinine was calculated from the results of the 4-hr PET. Laboratory values and 24-hr urine volume were also collected, and the values at baseline, 6 months and 12 months were averaged to determine the time-averaged values for variables.

### Statistical analysis

All parameters were described as the mean ± standard deviation for continuous variables and the percentage for categorical variables. Continuous data were compared using Student’s t-test to detect differences between two groups, and the chi-squared test or Fisher’s exact test was used for categorical variables. Univariable and multivariable linear regression analyses were used to determine the associations between TA-OH/ECW and echocardiographic parameters. The change in echocardiographic parameters from baseline to 12 months was assessed using a paired t-test. Univariable and multivariable binary logistic regression analyses were used to identify whether overhydration status had an influence on decreased EF at the end of study. A value of p < 0.05 was considered statistically significant. All analyses were performed using SPSS Statistics 21.0 (IBM Corporation, Armonk, NY, USA).
